# Exacerbation of colon carcinogenesis by *Blastocystis* sp.

**DOI:** 10.1371/journal.pone.0183097

**Published:** 2017-08-31

**Authors:** Vinoth Kumarasamy, Umah Rani Kuppusamy, Pailoor Jayalakshmi, Chandramathi Samudi, Nanthiney Devi Ragavan, Suresh Kumar

**Affiliations:** 1 Department of Microbiology, Faculty of Medicine, MAHSA University, Bandar Saujana Putra, Shah Alam, Malaysia; 2 Department of Biomedical Science, Faculty of Medicine, University of Malaya, Kuala Lumpur, Malaysia; 3 Department of Pathology, Faculty of Medicine, University of Malaya, Kuala Lumpur, Malaysia; 4 Department of Microbiology, Faculty of Medicine, University of Malaya, Kuala Lumpur, Malaysia; 5 Department of Biomedical Science, Faculty of Medicine, Mahsa University, Bandar Saujana Putra, Shah Alam, Malaysia; 6 Department of Parasitology, Faculty of Medicine, University of Malaya, Kuala Lumpur, Malaysia; University of Illinois at Chicago, UNITED STATES

## Abstract

Colorectal cancer (CRC) is one the most commonly diagnosed cancers worldwide and the number is increasing every year. Despite advances in screening programs, CRC remains as the second leading cause of cancer deaths in the United States. Oxidative stress plays an important role in the molecular mechanisms of colorectal cancer (CRC) and has been shown to be associated with *Blastocystis* sp., a common intestinal microorganism. In the present study, we aimed to identify a role for *Blastocystis* sp. in exacerbating carcinogenesis using *in vivo* rat model. Methylene blue staining was used to identify colonic aberrant crypt foci (ACF) and adenomas formation in infected rats whilst elevation of oxidative stress biomarker levels in the urine and serum samples were evaluated using biochemical assays. Histological changes of the intestinal mucosa were observed and a significant number of ACF was found in *Blastocystis* sp. infected AOM-rats compared to the AOM-controls. High levels of urinary oxidative indices including advanced oxidative protein products (AOPP) and hydrogen peroxide were observed in *Blastocystis* sp. infected AOM-rats compared to the uninfected AOM-rats. Our study provides evidence that *Blastocystis* sp. has a significant role in enhancing AOM-induced carcinogenesis by resulting damage to the intestinal epithelium and promoting oxidative damage in *Blastocystis* sp. infected rats.

## Introduction

Cancer is a concern for human health worldwide. It is one of the most aggravating diseases and bears the threat of mortality. It begins with the transformation of a normal cell into a cancerous cell and occurs through many stages over a number of years. Colorectal cancer (CRC) is one of the most commonly diagnosed cancers worldwide and is a major cause of cancer mortality [1]. The common signs of colorectal cancer include bloody stool and weight loss [2]. Increased intake of red meat and consumption of alcohol is known to increase the risk for CRC besides genetic factors [3,4]. Besides, patients who suffer from inflammatory bowel disease for long period time are prone to get CRC [5]. In Malaysia, CRC is the commonest cancer to affect both men and women [6]. Despite the availability of good treatment protocols, it continues to be the third highest cause of cancer mortality, largely because patients are diagnosed at a late stage. The molecular basis of individual susceptibility to colorectal cancer and factors responsible for the development of cancer are not well established. Intestinal pathogens such as bacteria and parasites have also been shown to play a role in contributing to CRC. There has been extensive research carried out on infections and cancer ever since the 1980s. About 18% of all cancers worldwide have been associated with infectious agents [7].

*Blastocystis* sp. is one the most common parasites found in the gastrointestinal tract of humans and animals [8]. The organism exists in multiple forms and is transmitted through the fecal-oral route [9]. The pathogenicity of *Blastocystis* sp. have been related to several chronic conditions such as acquired immune deficiency syndrome (AIDS) [10] and colorectal cancer (CRC) [11]. *Blastocystis* sp. Has a higher prevalence in developing countries compared to developed countries. High prevalence of *Blastocystis* sp. in developing countries is commonly associated with poor hygiene and close contact with animals [12, 13]. The symptoms caused by *Blastocystis* sp. are non-specific including diarrhoea, abdominal pain, nausea, anorexia, vomiting, weight loss and flatulence [12, 13]. The opportunistic nature of *Blastocystis sp*. was revealed when the parasites were confirmed in CRC patients during chemotherapy treatment [14]. Besides, high levels of oxidative damage in *Blastocystis sp*. infected rats and positive correlation amongst lipid and protein damage with hydrogen peroxide levels was reported previously [15].

Pathogenicity of *Blastocystis* sp. is still argumentative and non-conclusive. A few findings showed that only certain subtypes of *Blastocystis* sp. cause symptoms. For instance subtype 3 *Blastocystis* sp. is known to be pathogenic strain in Malaysia [16], Singapore [17] and USA [18]. Previously we reported that solubilized antigen isolated from *Blastocystis* sp. has the ability to suppress peripheral blood mononuclear cell while promoting the proliferation of human colorectal cells [19]. A comparison study carried out in our laboratory showed that *Blastocystis* sp. antigen from symptomatic patients triggered an increase in the proliferation of colorectal cancer cells compared to that of asymptomatic patients [20]. Antigen isolated from subtype 3 *Blastocystis* sp. showed the most prominent effect towards the proliferation of colorectal cancer cells [21]. *Blastocystis* sp. was shown previously to have detrimental effects towards the intestinal epithelial cells via apoptosis and protein disintegration. The pathogenic effect of *Blastocystis* sp. was further proven with the evidence of the formation of cysteine proteases [22]. In another study, intestinal cells were found to exhibit low level of IgA and increased level of pro-inflammatory cytokines in the presence of *Blastocystis* infection [23, 24]. Even though numerous *in vitro* studies have been carried out to associate *Blastocystis* with colorectal cancer, there were no studies conducted thus far using cancer-induced rat models to validate this association.

Oxidative stress is a normal phenomenon in the body. However, oxidative stress that persists for long period of time could lead to many pathophysiological conditions which results in diseases including cancer [25]. Oxidative stress occurs when the formation of reactive oxygen is greater than the body's ability to detoxify the reactive intermediates. Studies have shown that oxidative stress is elevated in humans infected with intestinal parasites such as *Blastocystis* sp. in cancer patients [14]. A previous study showed that the pathogenicity of *Blastocystis* sp. infection is more prominent when stress is present in rats [15]. Various studies have also reported on the presence of oxidative stress in serum and liver tissues of subjects infected with other parasites [26,27]. However there has been no study carried out to assess the oxidative stress levels in cancer induced and *Blastocystis* sp. infected rats thus far.

To date, this is the first study to evaluate the pre-neoplastic changes and the formation of aberrant crypt foci (ACF) in the colon of *Blastocystis* sp. infected rats associating the oxidative stress parameters which include protein oxidation and lipid peroxidation. Azoxymethane (AOM), a potent carcinogen which is commonly used to induce colon cancer in rats and mice was used in this study to induce carcinogenesis as it resembles the spontaneously forming colorectal carcinoma in humans [28]. It has been widely used in studies evaluating efficacy of preventative treatment of cancer [29, 30].

## Materials and methods

### *Blastocystis* sp. cysts isolation

Faecal sample was obtained from an asymptomatic individual who was having subtype 3 *Blastocystis* sp. determined via polymerase chain reaction (PCR) technique [31]. Ficoll-Paque density gradient centrifugation method was carried out to isolate *Blastocystis* sp. cyst from fresh faecal samples [32]. The faecal sample was re-suspended in phosphate buffered saline (PBS) and the faecal concentrate (4ml) was subsequently layered on 5ml of Ficoll-Paque and centrifuged at 1,600 x g for 20 minutes. *Blastocystis* sp. cyst layer formed upon centrifugation was transferred into a sterile Falcon tube, re-suspended in 1ml of PBS and washed 2 to 3 times with PBS containing 1% penicillin-streptomycin to eliminate bacterial contamination. Purified cysts were then counted using the trypan-blue method prior to inoculating them into rats.

### Experimental animals and treatments

**Fig 1:**
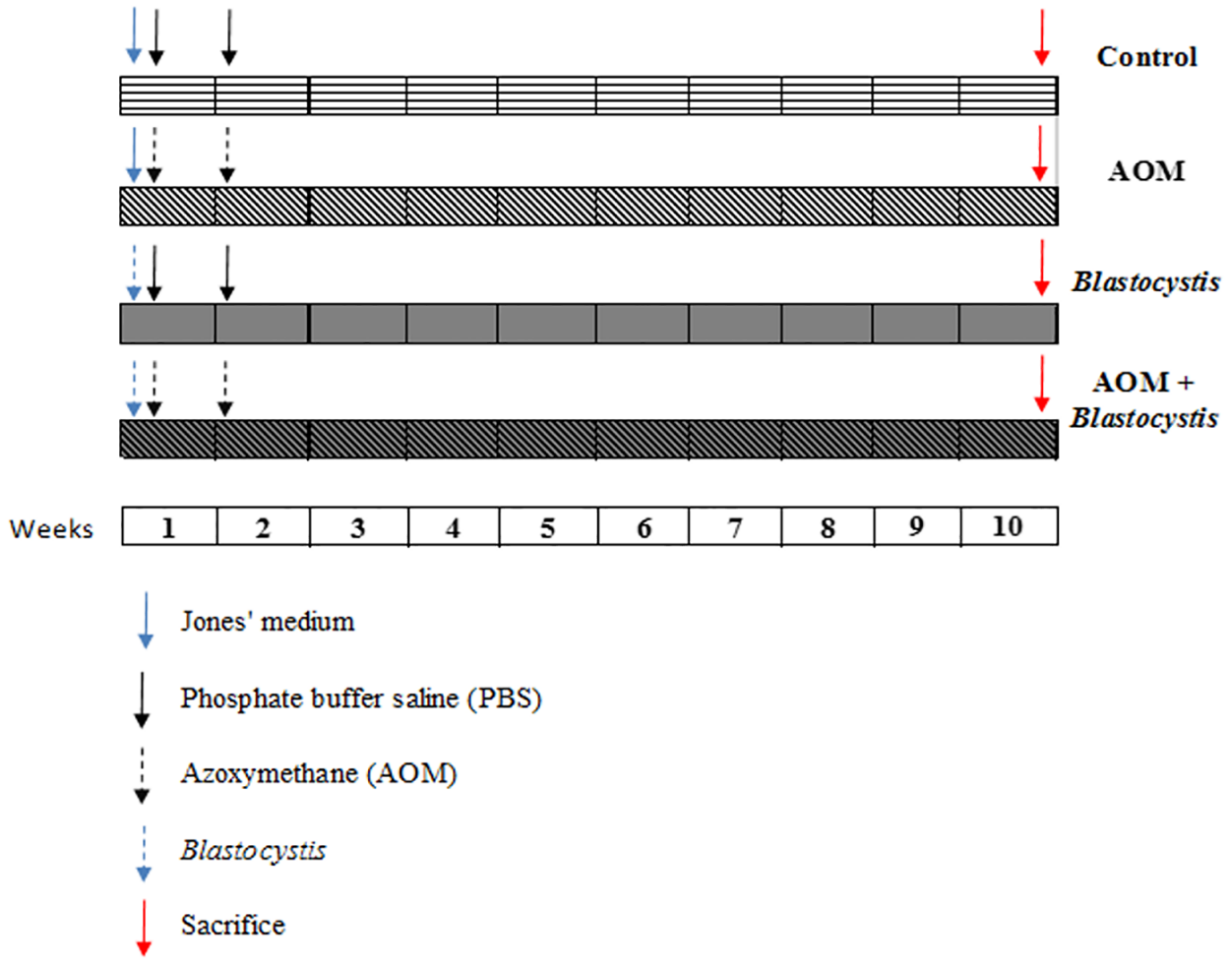
Experimental design for *in vivo* studies.

Three-weeks-old male Wistar rats (n = 24) with a mean weight of 65g were separated into 4 groups consisting of 6 rats per group and treated as follows for the entire duration of ten weeks of the study: control group (untreated) was administered with 0.3ml Jones’ medium by the oral route followed by PBS injection intraperitoneally once a week for two weeks, second group was administered with 0.3ml Jones’ medium by oral route followed by 15mg/kg azoxymethane (AOM) (Sigma-Aldrich, Saint-Quentin Fallavier, France) injection intraperitoneally once a week for two weeks, third group was inoculated with *Blastocystis* sp. cyst (ten thousand cysts/ ml sterile saline) orally followed by PBS injection intraperitoneally once a week for two weeks and the last group was inoculated with *Blastocystis* sp. cyst (ten thousand cysts / ml sterile saline) orally followed by 15mg/kg AOM injection intraperitoneally once a week for two weeks. The concentration of *Blastocystis* sp. cyst and AOM used in this study was based on a pervious study by Yoshikawa et al. (2004) [33] and Gosse et al. (2005) [34] respectively. All the rats were sacrificed on the 10^th^ week. The experimental design involving the inoculation of rats with *Blastocystis* sp. and treatment with AOM has been illustrated (Fig 1). Animals were caged individually under standardized conditions (22°C, 60% relative humidity, 12 h light/12 h dark cycle, 20 air changes/h). They were fed with standard diets and water was given *ad libitum*, body weights were recorded once a week. Permission to perform all animal experiments was given by the University Malaya Animal Ethics Committee. Care was taken to minimize the numbers of animals used in this experiment in accordance with the ARRIVE guidelines (S1 Checklist).

### *Blastocystis* sp. screening

The rat faecal samples were screened every day for the presence of *Blastocystis* sp. post-inoculation until the 16th day and thereafter, once a week until they were sacrificed. Faecal samples obtained were cultured in Jones’ medium supplemented with 10% horse serum and incubated at 37°C and then screened after 24h or 48h for the parasite.

### Histopathological processing and assessment of aberrant crypts in the rat colon

The tissues (small intestine, caecum, large intestine and rectum) fixed in 10% formalin were cut into pieces of approximately 3–4 mm size and processed for Hematoxylin and Eosin (H & E) Stain. Aberrant crypts quantification was carried out on a 6 cm segment of the distal part of the colon. The segment after washing with saline was then cut open, pinned flat and fixed in 10% formalin. The colon was stained with 0.2% methylene blue for 5 min and then rinsed in Krebs-Ringer buffer [35, 36]. It was then placed onto a glass slide and examined microscopically using a low power objective for assessment of the number of crypts. Aberrant crypts were identified based on three criteria namely i) dilated irregular luminal openings ii) thicker epithelial lining iii) an increased pericryptal zone relative to normal crypts.

### Urine and blood sample collection for biochemical analysis

Urine and blood samples from all rats were collected early morning prior sacrifice by placing them in a closed cylindrical jar with diethyl ether to ensure the absence of righting reflex. Blood samples were the immediately collected and processed. Blood was drawn via cardiac puncture whilst rats were under general anaesthesia. It was allowed to clot for 15 min at room temperature and serum was separated by centrifugation at 1500 ×*g* for 10 min.

### Biochemical assays

Lipid hydroperoxide (LHP) level was determined as it reacts with 1-methyl-2-phenylindole (MPI) under acidic conditions to form blue-coloured chromophore which was measured spectrophotometrically [37]. Advanced oxidation protein products (AOPP) formation was observed by the action of chlorinated oxidants which could be measured spectrophotometrically [38]. The AOPP concentrations were calculated based on the standard curve prepared using chloramine T and the results were expressed as mmol/l of chloramine T equivalents. The level of hydrogen peroxide (H_2_O_2_) was determined based on the oxidation of ferrous ion by oxidizing agent (in the samples) into ferric ions [39]. The amount of ferric product was measured as a xylenol orange complex at 560 nm. Ferric reducing antioxidant power (FRAP) assay was used to measure non-enzymatic antioxidants or reductants in the sample [40]. Antioxidants in the sample would reduce ferric ion tripyridyltriazine (Fe^2+-^TPTZ) to ferrous ion tripyridyltriazine (Fe^3+^-TPTZ) at low pH. The resulting blue-coloured ferrous-tripyridyltriazine complex was measured spectrophotometrically. Determinations of reductant concentration were done based on the standard curve of ferrous sulphate heptahydrate (FeSO_4_.7H_2_O) and values were expressed as μmol/l.

### Statistical analysis

All data were analysed using SPSS version 22. Values are expressed as mean ± S.E.M. and the significant difference between the groups were analysed using Student’s t-test. Correlations between the parameters for both control and parasite infected subjects were identified by Pearson’s correlation coefficients test and differences were considered significant when P < 0.05.

## Results

### Stool examination

*Blastocystis* sp. infected rats showed a lower body weight compared to uninfected rats (controls) (P<0.05) (Table 1). This difference in weight gain was evident starting from week 2 post-inoculation. Rats infected with *Blastocystis* sp. and injected with AOM showed a higher weight gain compared to rats without AOM injection (P<0.05). Stools from AOM-rats with *Blastocystis* sp. infection generally were found to be softer and watery compared to that of AOM-rats without *Blastocystis* sp. infection. Stool samples of control rats showed total absence of *Blastocystis* sp. throughout the period of investigation implying that there was no possibility of contamination from external sources such as food and water. *Blastocystis* sp. was present in the stool of all other infected rats from Day 3 to Day 7 post-inoculation. The parasite was present at irregular intervals from Day 8 post-inoculation onwards till the day of sacrifice.

**Table 1:** Body weight (in grams) of uninfected rats (controls), *Blastocystis* infected, AOM-treated, AOM + *Blastocystis* (co-infection) rats before inoculation, post-inoculation and before sacrifice.

Groups	Weight, g		
	Before Inoculation (Day 1)	Post inoculation (Day 30)	Before sacrifice (Day 65)
Control, n = 6	65 ± 2.7	201 ± 5.7	560 ± 6.6
*Blastocystis*, n = 6	65 ± 3.3	166 ± 9.1	450 ± 8.4
AOM, n = 6	65 ± 3.5	160 ± 7.2	420 ± 8.2
AOM + *Blastocystis*, n = 6	65 ± 4.2	175 ± 7.5	415 ± 9.1

Data are expressed as mean ± SD

### Histological examination

**Fig 2:**
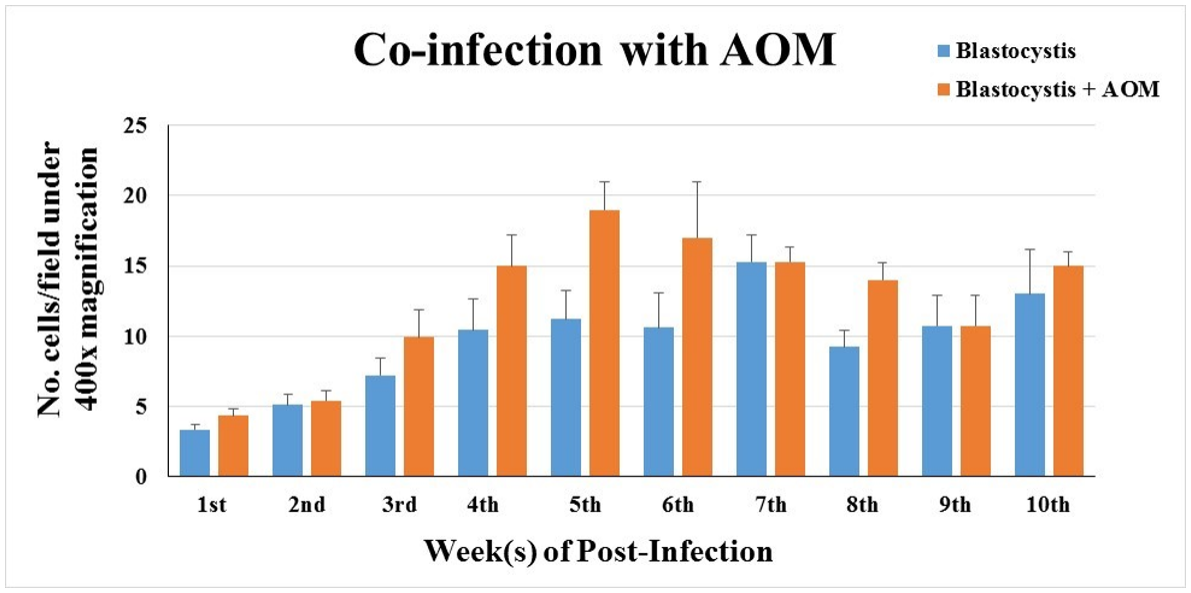
Parasite burden in stool samples of AOM-rats co-infected with *Blastocystis* according to weeks of infection. Data expressed in mean ± SD.

**Fig 3:**
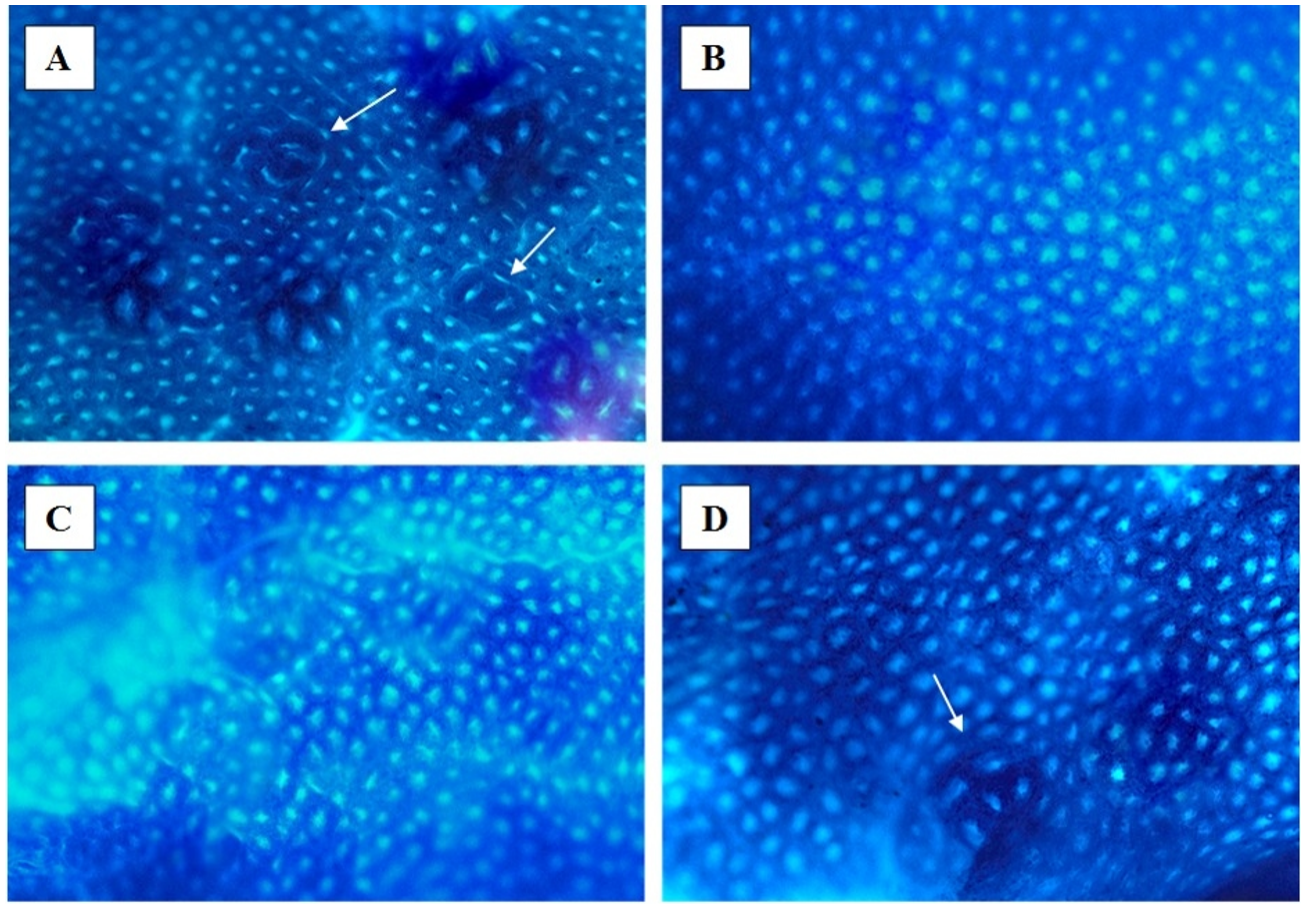
Distal colon tissue stained with methylene blue by trans illumination and surface examination in the inverse light microscope (x 40 magnification). (A) AOM + Blastocystis; (B) Control; (C) Blastocystis; (D) AOM.

**Fig 4:**
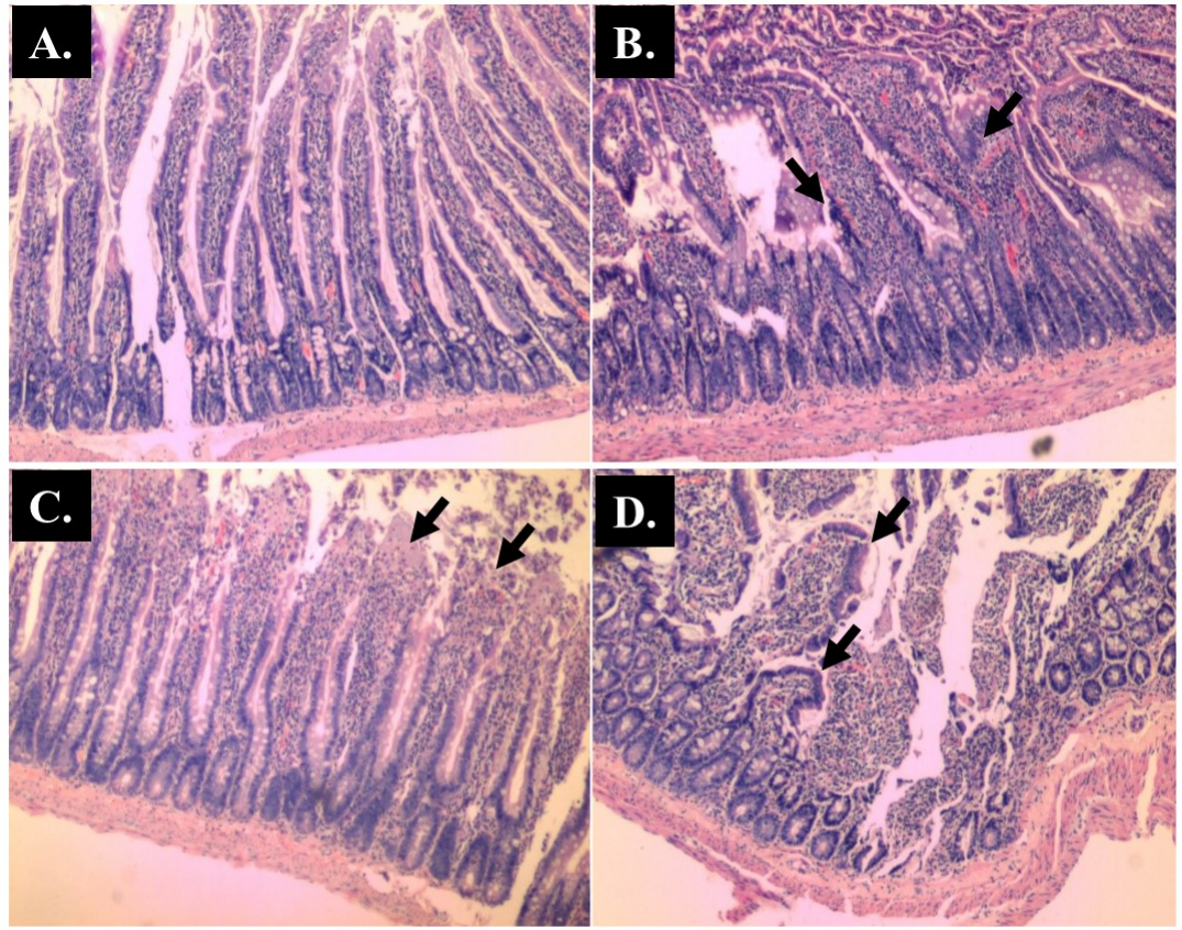
Representative histological Hematoxylin and Eosin (H and E) staining of villi of small intestine (x 40 magnifications). Control; (B) AOM; (C) *Blastocystis*; (D) AOM + *Blastocystis*.

**Fig 5:**
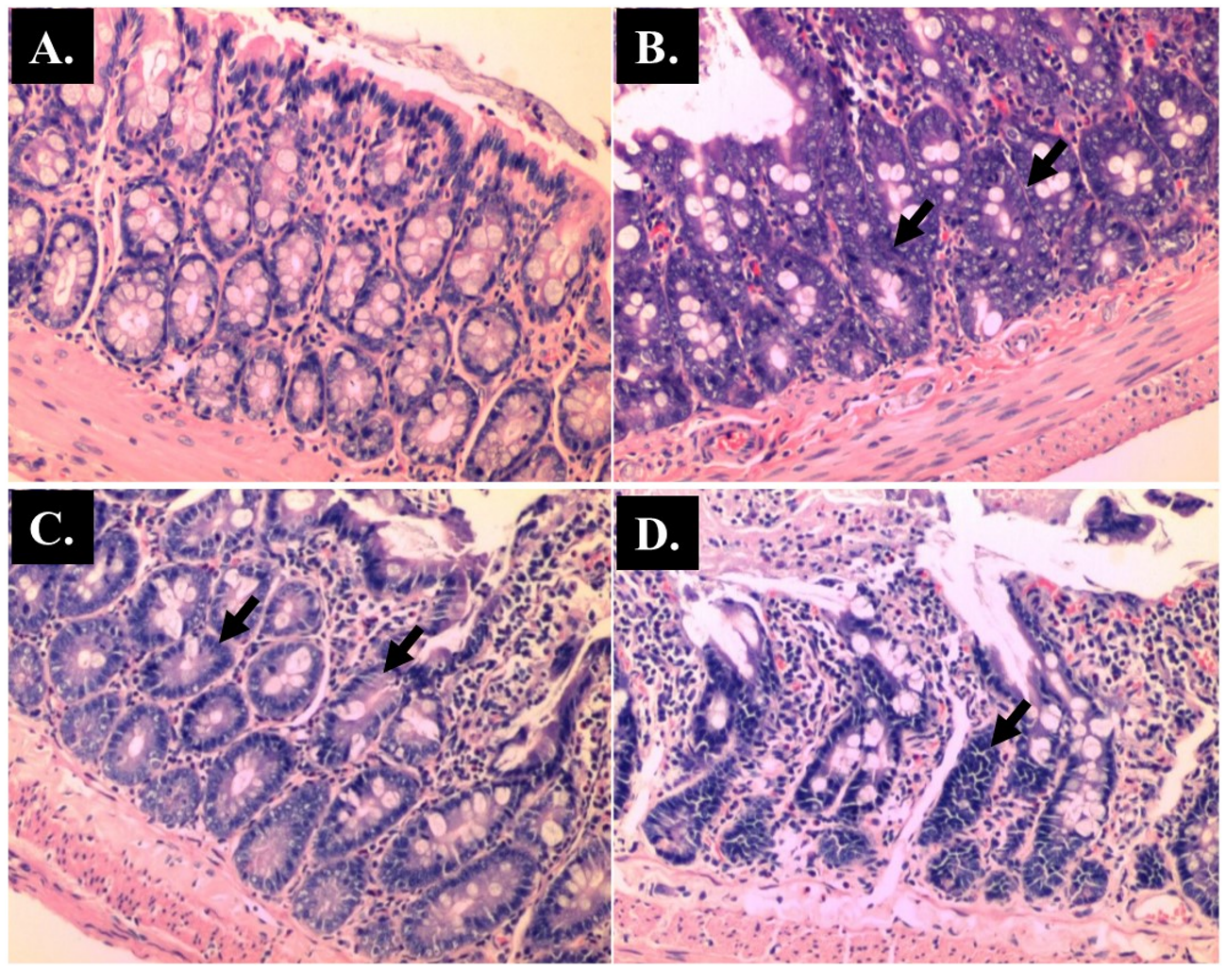
Representative histological Hematoxylin and Eosin (H and E) staining of goblet cells of small intestine (x 100 magnifications). Control; (B) AOM; (C) *Blastocystis*; (D) AOM + Blastocystis cyst.

**Fig 6:**
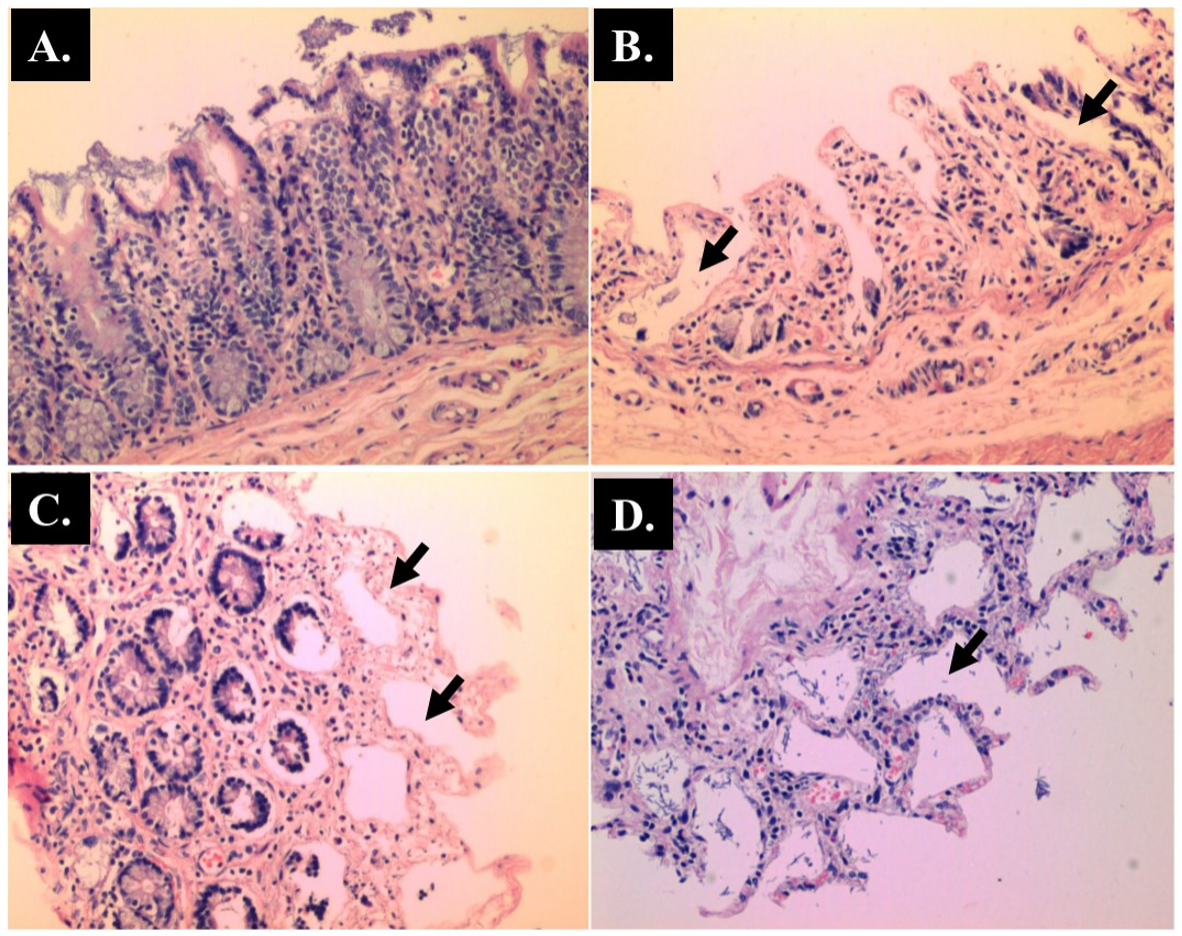
Representative histological Hematoxylin and Eosin (H and E) staining of caecum. (A) Control(x 40 magnifications); (B) AOM (x 40 magnifications); (C) Blastocystis (x 100 magnifications); (D) AOM + *Blastocystis* cyst (x 100 magnifications).

**Fig 7:**
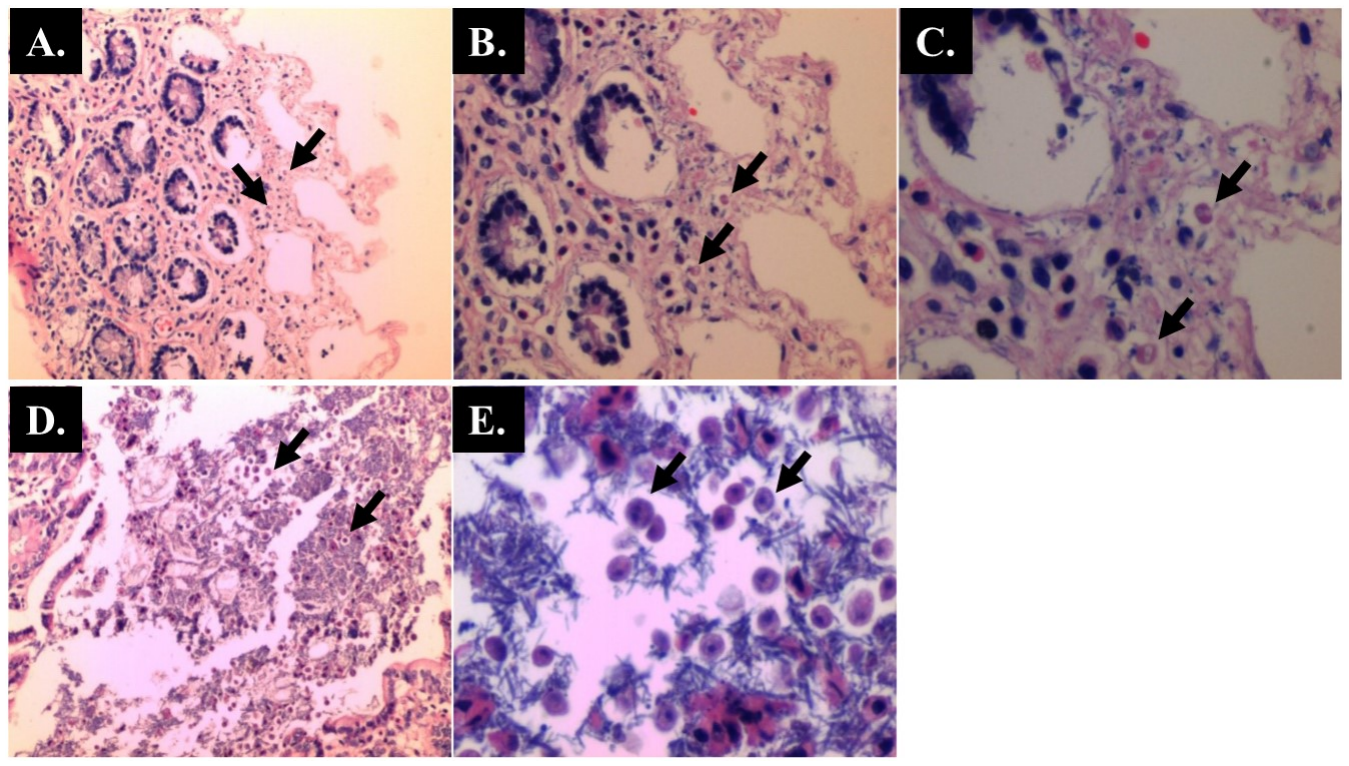
Caecum of AOM-rats with *Blastocystis* infection (Hematoxylin and Eosin staining) showing numerous villous enterocytes with *Blastocystis*-like organisms. (A & D) x 40 magnifications; (B) x 100 magnifications; (C) x 200 magnifications; (E) x 400 magnifications.

**Fig 8:**
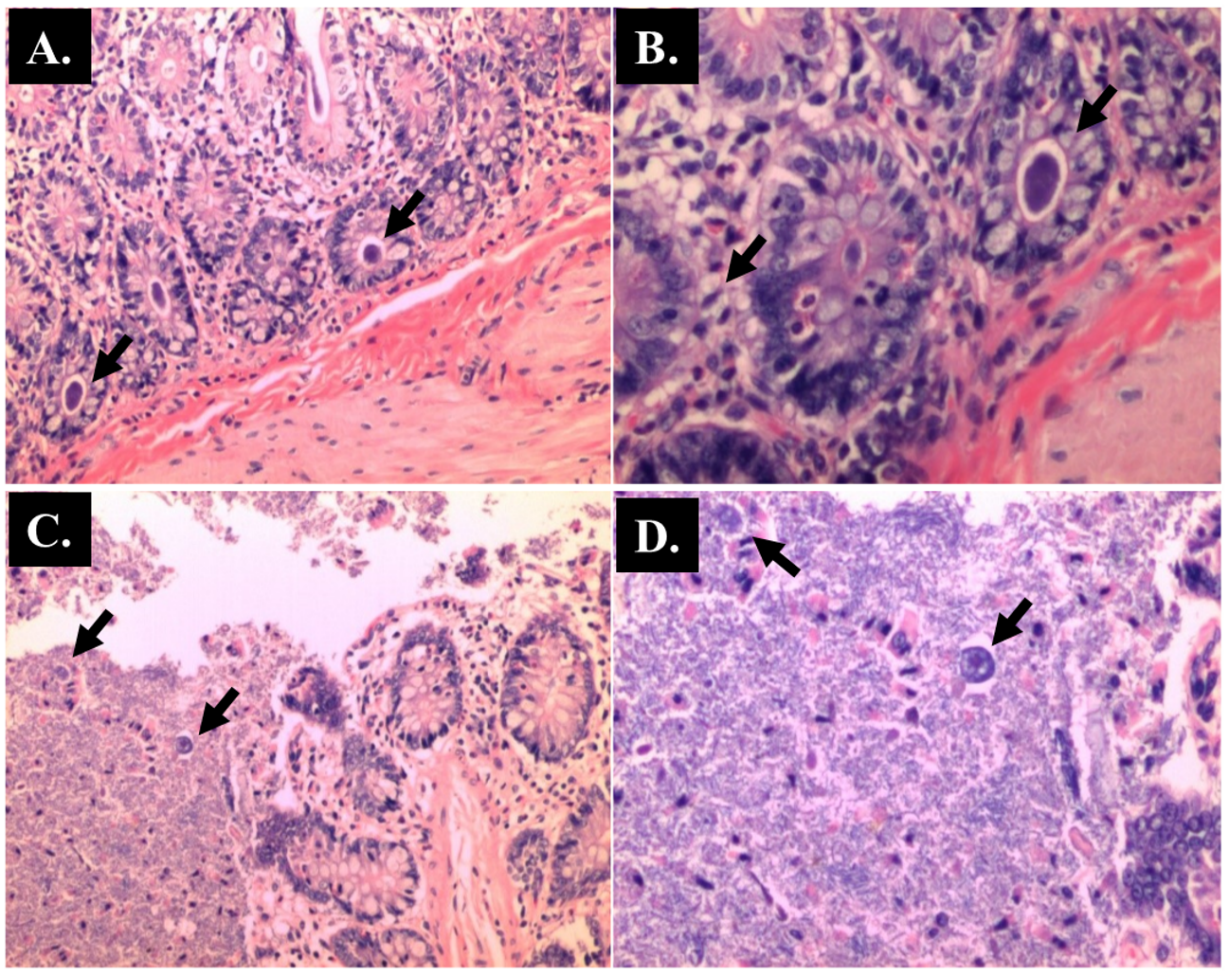
Villous enterocytes contain *Blastocystis*-like organisms (Hematoxylin and Eosin staining). (A) x 40 magnifications; (B) x 200 magnifications; Lumen (C and D) (x 40 magnification).

**Fig 9:**
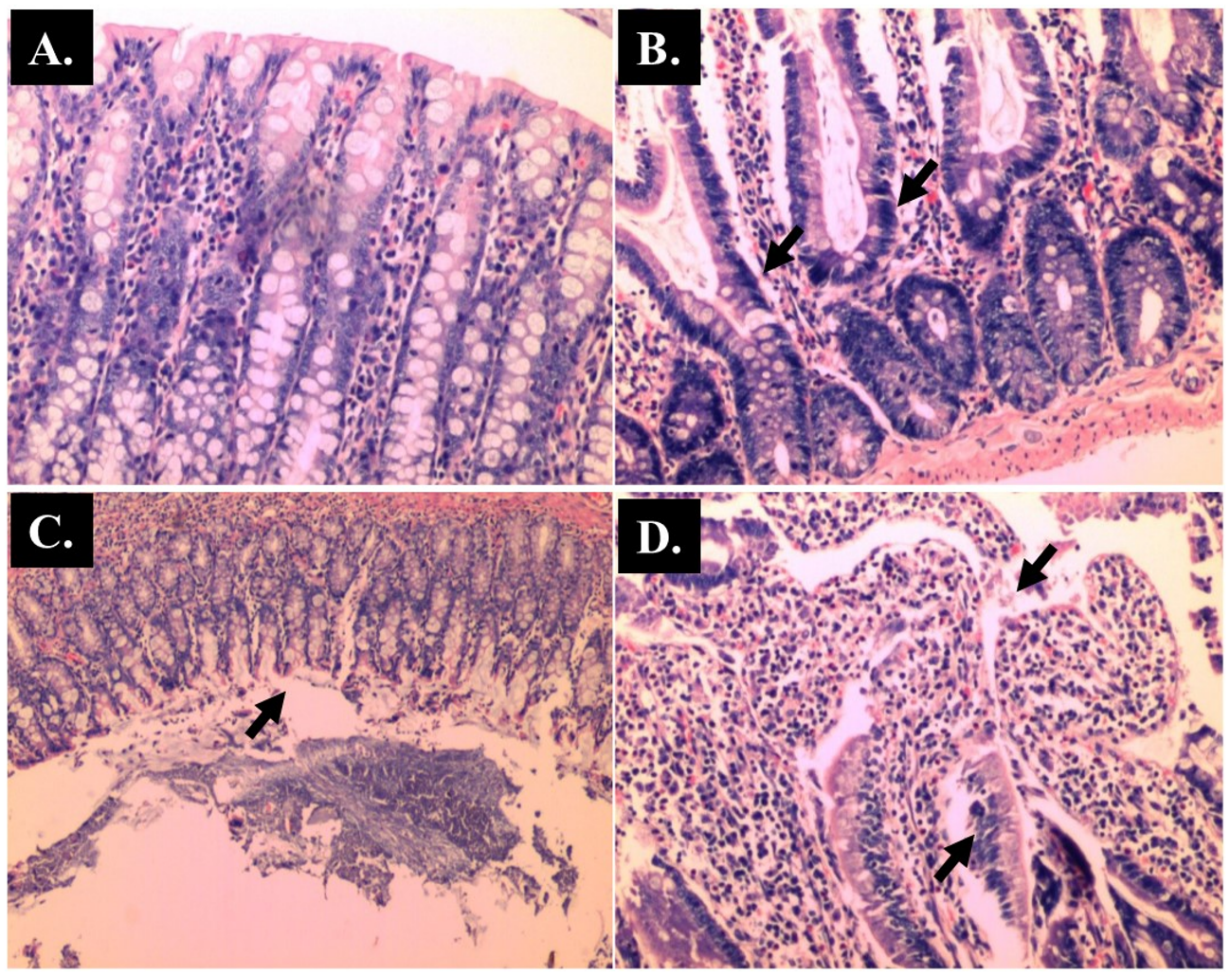
Representative histological Hematoxylin and Eosin (H and E) staining of large intestine. (A) Control (x 100 magnifications); (B) AOM (x 100 magnifications); (C) *Blastocystis* (x 20 magnifications); (D) AOM + *Blastocystis* (x 40 magnifications).

**Fig 10:**
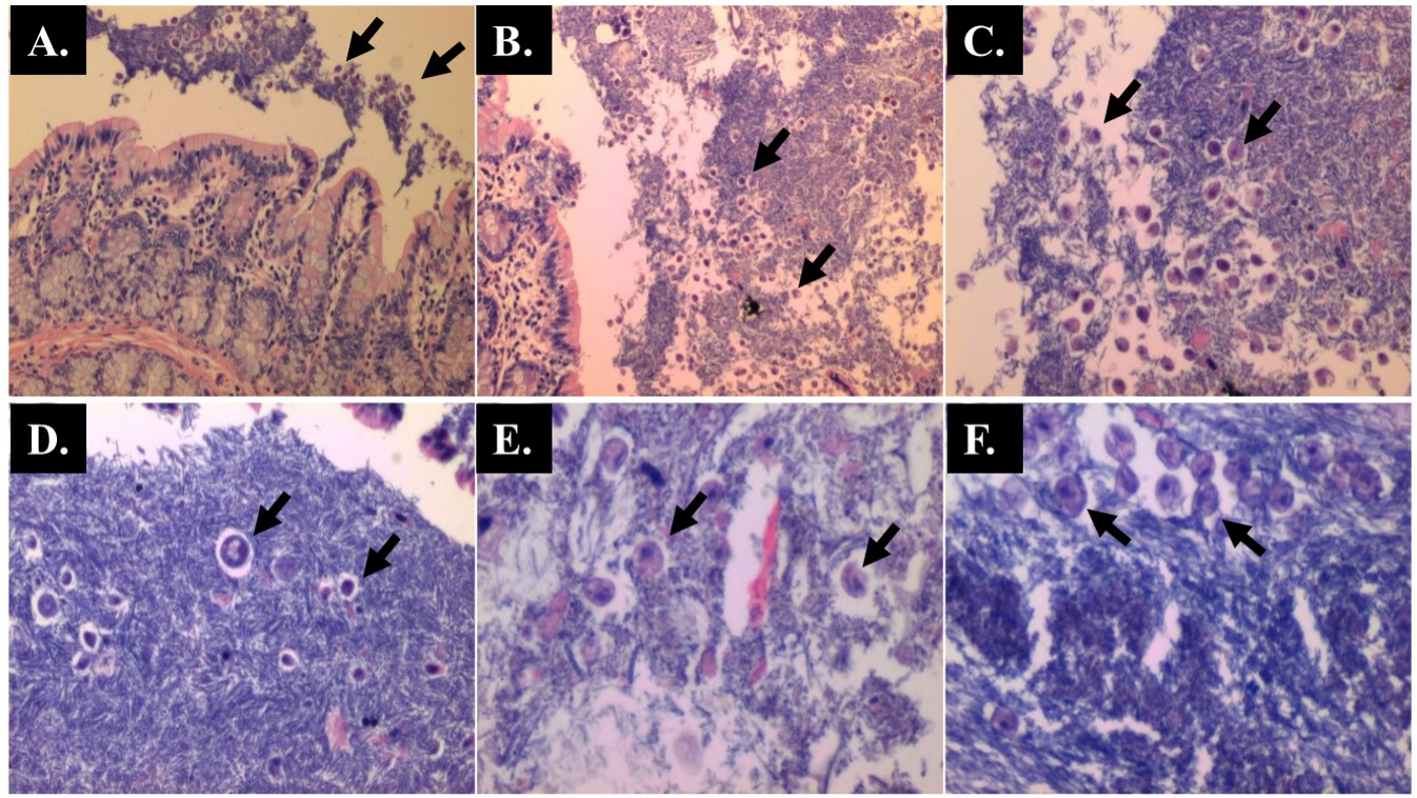
Small intestine of AOM-rats with *Blastocystis* infection shows the lumen contains *Blastocystis*-like organisms. (A & B) x 40 magnifications; (C) x 100 magnifications; (D, E & F) x 200 magnifications.

**Fig 11:**
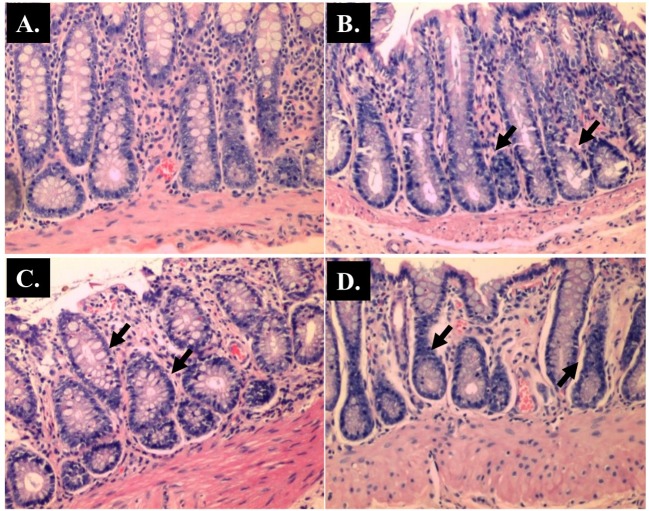
Representative histological Hematoxylin and Eosin (H and E) staining of rectum. (A) Control (x 100 magnifications); (B) AOM (x 100 magnifications); (C) *Blastocystis* (x 100 magnifications); (D) AOM + *Blastocystis* (x 100 magnifications).

The number of *Blastocystis* sp. (vacuolar form) and cysts per field under 400x magnification using direct microscopic examination from the first week of post-inoculation kept increasing until the day of sacrifice (Fig 2). All rats injected with AOM developed numerous abnormal and hyperplastic colonic crypts, regardless of treatment (Fig 3). The AOM-rats co-infected with *Blastocystis* sp. had 38 ± 5 ACF per cm on average compared to AOM-controls which had 24 ± 4 ACF per cm (Table 2). The co-administration of *Blastocystis* sp. cyst resulted in a 1.6-fold increase in the number of crypts when compared with control rats treated with AOM only. Two of the co-*Blastocystis* sp. infected AOM-rats were found to have adenomas incidence. Gross pathological changes were prominently seen in the *Blastocystis* sp. infected rats with slight hyperaemia observed in some of them. The histopathological changes were observed in different parts of the intestinal tract in all the cohort groups compared to controls (Figs 4–11).

**Table 2:** Effect of *Blastocystis* on AOM-induced ACF and adenomas formation in the colon of AOM treated Wistar rats as well as AOM and *Blastocystis* co-infected Wistar rats.

Groups	ACF Incidence	ACF multiplicity/cm	Adenomas incidence	No. of adenomas/colon
AOM	6/6.	24 ± 4	0/6	0
AOM + *Blastocystis*	6/6.	38 ± 5*	2/6.	2.5 ± 3

Values shown are mean values ± SD

*Significantly different from AOM group by Student’s t—test (P <0.01)

The histopathological sections of distal colon tissue of the intestinal tract were compared between infected and control rats (Fig 4). Gross pathological changes were evident in the infected rats. Aberrant crypts foci were observed in AOM-controls and *Blastocystis* sp. infected AOM-rats. In the small intestine of *Blastocystis* sp. infected rats, slight sloughing of the mucosal epithelium was observed at the tip of the villi facing the luminal surface (Fig 4C). Similar results were also observed in the other parts of the intestinal tract of *Blastocystis* sp. infected rats such as caecum (Fig 6C), large intestine (Fig 9C), and rectum (Fig 11C). The goblet cells were irregular, large in size, and reduced in number (Fig 5C). Tubular adenoma with very mild dysplasia was observed in rats injected with AOM (Fig 4B). Major dysplasia and the presence of hyperplastic aberrant crypts were observed in rats injected with AOM and co-infected with *Blastocystis* sp. (Fig 4D). The caecum of *Blastocystis* sp. infected rats showed significant sloughing of the mucosal epithelium and the goblet cells were reduced in numbers (Fig 6C). AOM injected rats with *Blastocystis* sp. infection showed a few areas in the lamina propria to have infiltration of the mucosa with polymorphonuclear neutrophils and monocytes with the presence of early pre-neoplastic lesions (Fig 6D). This extended up to the submucosa and diffuse lymphoid follicular hyperplasia was observed in some areas of mucosa and submucosa. The goblet cells in the colon were irregular in shape, enlarged and reduced in number. We also observed *Blastocystis*-like organism in the mucosal layer of caecum (Figs 7 and 8) of *Blastocystis* sp. infected AOM-rats. Many *Blastocystis*-like organisms were also observed in the lumen of the caecum (Figs 7 and 8) and small intestine (Fig 10). Less severe changes were found in the large intestine of rats with AOM injection alone, which showed enlarged, irregular shaped goblet cells (Fig 9).

### Oxidative indices

Urinary levels of oxidative stress parameters were high in *Blastocystis* sp. infected AOM-rats compared to the AOM controls. The levels of LHP, AOPP, H_2_O_2_ and FRAP were positively correlated with each other in *Blastocystis* sp. infected rats and uninfected rats (normal) (Table 3) as well as AOM-group compared to the AOM-controls (Table 4). For example, AOPP levels and H_2_O_2_ levels exhibited a positive correlation (P < 0.001) in *Blastocystis* sp. infected group, while it was non-significant in AOM-controls.

**Table 3:** Pearson’s correlation between the urinary biochemical variables in normal and *Blastocystis* infected group (control experiment for AOM + co-infection with *Blastocystis*).

**Normal**				
	AOPP	LHP	H_2_O_2_	FRAP
AOPP	1			
LHP	0.6751***	1		
H_2_O_2_	0.6144**	0.5123***	1	
FRAP	NS	NS	NS	1
***Blastocystis***				
	AOPP	LHP	H_2_O_2_	FRAP
AOPP	1			
LHP	NS	1		
H_2_O_2_	0.7220***	0.6543***	1	
FRAP	0.654***	0.6601**	NS	1

Asterisks indicate significant correlation at different levels of 0.01(**) and 0.0001 (***); NS, not significant.

**Table 4:** Pearson’s correlation between the urinary biochemical variables in AOM-treated controls and rats treated with AOM + co-infection with *Blastocystis*.

**AOM**				
	AOPP	LHP	H_2_O_2_	FRAP
AOPP	1			
LHP	0.7581***	1		
H_2_O_2_	NS	NS	1	
FRAP	NS	NS	NS	1
**AOM + *Blastocystis***				
	AOPP	LHP	H_2_O_2_	FRAP
AOPP	1			
LHP	0.7112***	1		
H_2_O_2_	0.8121***	0.5332**	1	
FRAP	0.6723***	0.5501**	0.8621***	1

Asterisks indicate significant correlation at different levels of 0.01(**) and 0.0001 (***); NS, not significant.

**Fig 12:**
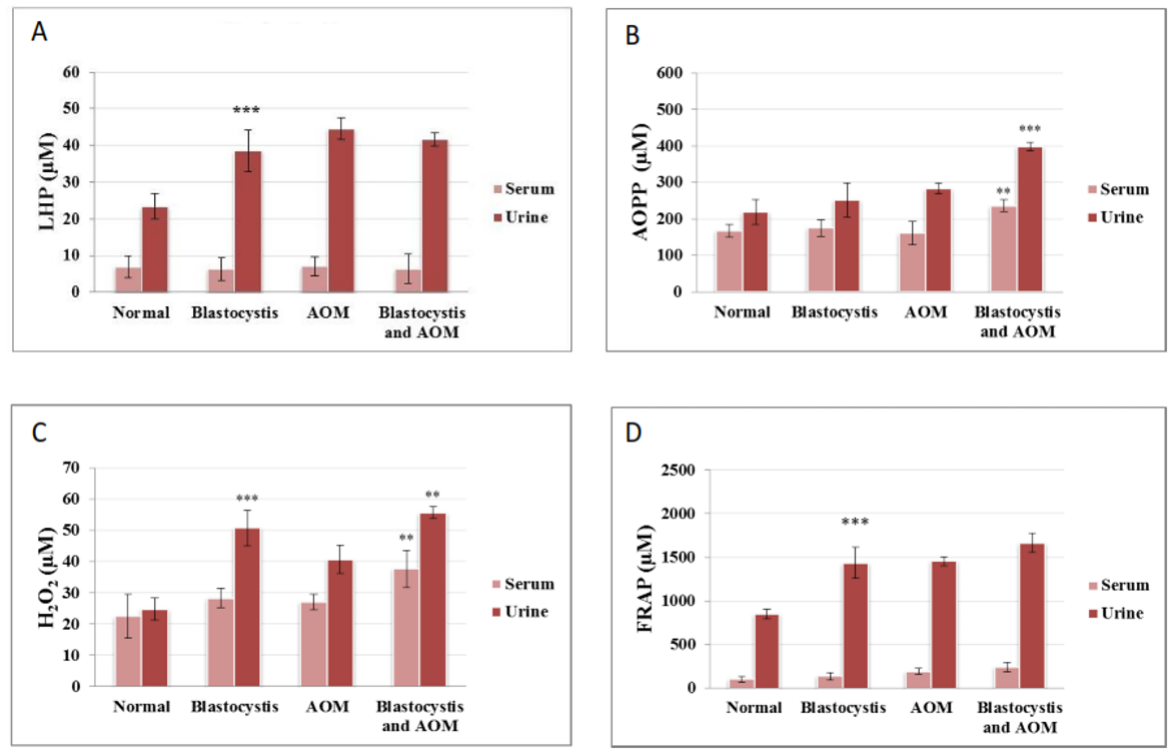
Comparison of (A) LHP, (B) AOPP, (C) H_2_O_2_ and (D) FRAP levels in blood and urine samples of normal, *Blastocystis* infected, AOM-treated controls and rats treated with AOM + *Blastocystis* infection. Groups of six rats were inoculated with Blastocystis and injected with AOM simultaneously (co-infection). Data are given as mean SEM of six animals/group by Student’s t-test (SPSS version 13). ***P<0.001 is the comparison between columns of Blastocystis and normal as well as Blastocystis + AOM and AOM.

Generally AOM-rats with *Blastocystis* sp. infection, showed higher levels of oxidative damage especially lipid peroxidation (LHP) and protein damage (AOPP) compared to their respective AOM-controls (Fig 12A and 12B). AOPP in plasma and serum have been widely used as a marker of free radicals induced protein damage in renal disease and diabetic complications [41]. To date, this is the first study demonstrating the level of serum AOPP in rats induced with AOM. In line with this observation, a previous study has reported that the plasma AOPP level was higher in the CRC patients than in normal subjects [39]. High level of antioxidants (reflected by high FRAP level) was observed in AOM-rats with *Blastocystis* sp. infection compared to normal controls (Fig 12D).

## Discussion

Numerous reports show that *Blastocystis* sp. is found in individuals who show gastrointestinal symptoms as well as in asymptomatic individuals [42, 43, 44]. On the basis of these findings, researchers have dismissed *Blastocystis* sp. to be non-pathogenic continuing the enigma on the parasite’s role and its pathogenic status. However, the identification of potential virulence factors such as cysteine proteases and the presence of numerous *in vitro* and *in vivo* findings show its pathogenic potential in humans [45, 46, 47]. Moreover various studies have also shown the association between its subtype variation and pathogenicity.

In the present study, we used AOM to induce aberrant crypt formation in *Blastocystis* sp. infected Wistar rats. AOM is a carcinogenic chemical known to induce intestinal adenomas and adenocarcinomas in rats [48]. The development of colon cancer involves a multi-stage process which involves the formation of aberrant crypts. Studies on the aberrant crypts have been widely used to understand the cause and exacerbation of colon cancer in rats and humans [49].

Our study was primarily carried out to establish the role of *Blastocystis* sp. infection in conditions relating to colorectal cancer. Previously, *Blastocystis* sp. infection has been shown in patients with colorectal adenoma but the incidence was reported higher in patients with Dukes’ A, B and C colorectal carcinoma [50]. *Blastocystis* sp. was speculated to trigger mutation in normal colonic epithelial cells leading to adenoma which could result in colorectal carcinoma due to other factors such as genetic [50]. Our study showed that *Blastocystis* sp. has the ability to enhance crypts formation when AOM is administered. Similar results were observed in rats infected with *Streptococcus bovis*, a species of bacteria which was associated with colorectal cancer in human [51]. However, *Blastocystis* sp. infection alone did not show the formation of colonic crypts implying that that the parasite may not form tumors but possess the potential to exacerbate existing ones.

In the present study, rats infected with *Blastocystis* sp. cysts showed a decrease in weight gain, compared to that of control rats. However, a significant decrease in weight gain was noticed in AOM-rats infected with *Blastocystis* sp. cysts compared to uninfected AOM-rats (P<0.05) (Table 1). Previously, reduced weight gain was observed in animals infected with other protozoans such as *Eimeria tenella* [52], *Cryptosporidium* [53] and *Sarcosystis spp*. [54]. Stools from infected rats were softer with mucus when compared to uninfected rats and lasted for 8 days post-inoculation after which the stools reverted to normalcy. The passing of soft stools implies mal-digestion, affecting the absorption of ingested food which could be due to reduced enzymatic digestion. The change in the stool consistency could be caused by the effect of enterotoxins produced by the vacuolar forms of *Blastocystis* sp. These enterotoxins could have stimulated active electrogenic chloride or bicarbonate secretion [55] which could result in malabsorption. The intestinal contents of caecum and colon in these rats showed the presence of *Blastocystis* sp. both by direct microscopy as well as when the respective contents were cultured *in vitro*.

We speculate that the increase in reactive oxygen species formation in the intestinal lumen is attributed to this mechanism. Damages caused by reactive oxygen species can lead to hypertrophy of the cells and deformity of the glands that may results in the ACF formation [56]. Therefore, histological examination was carried out to observe the pathogenic effect such as tissue invasion by the parasite and damages inflicted at the mucosal layer of the gastrointestinal tract.

In the present study, major dysplasia and hyperplastic aberrant crypts were seen mostly in the small intestine (Fig 4) and large intestine (Fig 9), and to a lesser extent in the caecum (Fig 6) and rectum (Fig 11). An inflammatory reaction could have been invoked as reported previously where the movement of fluid, plasma proteins, and leukocytes into tissues in response to injury, microbial invasion, foreign material, or antigens present in the secretory products of the parasite [57].

In the caecum, shedding of the epithelial cells was noticed at the tip of the villi. Large areas of the epithelial tissue of the mucosa in the caecum lost their structural integrity and became detached in AOM-rats with *Blastocystis* sp. infection. Therefore, it is likely that the decreased surface area produced in the sloughing of the mucosal epithelium subsequently led to the lowering of digestive and absorptive capabilities of the epithelial cells. Previously, infiltration of the lamina propria with inflammatory cells and sloughing of the mucosal epithelium was observed in the caecum infected with *Blastocystis* sp. whereby the parasite was administered into experimental mice via intramuscular injection [58].

Greater surface area of the mucosa was affected in *Blastocystis* sp. infected rats injected with AOM as compared to untreated rats. In these rats, sloughing of the mucosal cells was severe and hyperplasia of the goblet cells was also more prominent, indicating that these pathological changes are more exacerbated when rats with both carcinogen and the parasite exist together. The level of parasite excretion in the stools was shown to increase as cancer progresses which reflect the opportunistic nature of the parasite as described in a previous study [59].

Pathological changes were more pounced in *Blastocytsis* sp. infected rats with carcinogen compared to *Blastocystis* sp. infected rats without the injected AOM. Nevertheless, *Blastocystis* sp. infection alone did not produce significant dysplasia in the rats. The lesions produced by *Blastocystis* sp. infection including sloughing of the mucosal layer and inflammation of the lamina propria. These changes consequently resulted in the mal-digestion and malabsorption, thus leading to diarrhoea and hence a decrease in weight gains of the infected rats as mentioned earlier.

Oxidative stress has a crucial effect in colorectal tumorigenesis [60]. It is widely recognized as the key component in the pathogenesis of CRC complications. In the present study, a significant positive correlation existed among the levels of oxidative indices in the *Blastocystis* sp. infected AOM-group (Table 4). Increased lipid hydroperoxide level observed in the *Blastocystis* sp. infected rats (Fig 12A) indicates the presence of oxidative degradation of lipids in the cell membranes which can result in the cell damage. Reactive lipid peroxidation products have the ability to trigger the formation of DNA adducts which is directly associated with tumour development [61]. Elevated lipid peroxidation level has been shown previously in breast cancer patients [62]. Furthermore, biochemical studies carried out in animal host for other parasites such as *Giardia lamblia* and *Microsporidia* sp. also showed an increased level of cell injury, lipid peroxidation and intestinal neutrophils accumulation [63].

The formation of AOPP (Fig 12B) and H_2_O_2_ (Fig 12C) is quantitatively elevated in this experiment and it shows significantly higher levels in *Blastocystis* sp. co-infected with AOM. Oxidation of proteins have been found to have a significant effect on the pathogenesis of cancers [64,65]. Besides, AOPP is usually elevated in patients with cancer where they were correlated with markers of oxidative stress [66, 67]. In CRC patients particularly, increased formation of protein carbonyl which is part of protein damage have been also reported [39,68]. The high level of H_2_O_2_ in *Blastocystis* sp. infected AOM-rats (Fig 12C) could be due to invasion of parasites that trigger the formation of free radical species such as nitric oxide (NO) and superoxide anion (O_2_^-^) by the host’s cellular immune response [58,59]. Inflammation process and the anti-microbial defence mechanism was found to be closely associated with oxidative stress in the liver, reported in a previous study in which elevated level of H_2_O_2_ was observed [69].

Free radicals formed from metabolic reaction in the host are normally removed by regulatory mechanisms involving antioxidants. Serum total antioxidant level was found to be very low as determined using FRAP assay. The high levels of urinary antioxidant activity in the infected group (Fig 12D) indicate that the host’s antioxidant regulatory system has been triggered to combat the oxidative damage caused by the increasing burden of parasitic infection [15]. Besides, the increase in FRAP levels in the parasite infected rats might be caused by an increase in serum uric acid due to biochemical changes that occur in response to the infection. For instance, plasma uric acid levels were reported to be high in mice with late stage *Plasmodium vinckei* infection [70]. Besides, uric acid is insoluble and has the ability to precipitate at high concentrations. These properties attribute to the accumulation of uric acid in developing eggs of roundworm and this was used in maintaining an osmotically constant surrounding in the water-impermeable eggs [71]. Hence, similar adaptation of such activity in *Blastocystis* sp. (vacuolar or cyst forms) infected rat is possible. A previous study has shown that increased plasma uric acid may result in about 60% of FRAP activity [72].

In conclusion, the present study clearly indicates *Blastocystis* sp. infection leads to disruption of normal gut function, resulting in damage to the intestinal mucosal layer and triggers oxidative stress which could contribute to the significant increase in crypts formation in AOM-treated rat models. The study establishes that *Blastocysis* sp. is a pathogen and there is a need to screen cancer patients for harbouring this parasite.

## Supporting information

S1 Checklist ARRIVE guidelines checklist. (PDF)
